# Pre-transplant donor HBV DNA+ and male recipient are independent risk factors for treatment failure in HBsAg+ donors to HBsAg- kidney transplant recipients

**DOI:** 10.1186/s12879-020-05704-1

**Published:** 2021-01-09

**Authors:** Xian-ding Wang, Shi-jian Feng, Jin-peng Liu, Tu-run Song, Zhong-li Huang, Yu Fan, Yun-ying Shi, Li-yu Chen, Yuan-hang Lv, Zi-lin Xu, Xiao-hong Li, Li Wang, Tao Lin

**Affiliations:** 1 Department of Urology/Institute of Urology, West China Hospital, Sichuan University, Number 37, Guoxue Alley, Chengdu, 610041 Sichuan China; 2 Organ Transplantation Center, West China Hospital, Sichuan University, Chengdu, Sichuan China; 3 Department of Nephrology, West China Hospital, Sichuan University, Chengdu, Sichuan China; 4 Department of Infectious Diseases, West China Hospital, Sichuan University, Chengdu, Sichuan China; 5 West China School of Clinical Medicine, Sichuan University, Chengdu, Sichuan China; 6 Department of Health Statistics, West China School of Public Health, Sichuan University, Chengdu, Sichuan China

**Keywords:** HBsAg+ living donors, Donor-derived HBV transmission, HBsAg- recipients, Kidney transplantation

## Abstract

**Background:**

In order to reduce the burden on organ shortage around the world, using potential infectious donor might be an option. However, scarce evidences have been published on kidney transplantation (KTx) from hepatitis B surface antigen (HBsAg) + donors to HBsAg- recipients [D (HBsAg+)/R(HBsAg-)] without hepatitis B virus (HBV) immunity. Here, we reported the results of D(HBsAg+/HBV DNA- or +)/R(HBsAg-) living KTx recipients with or without HBV immunity.

**Methods:**

We retrospectively identified 83 D(HBsAg+)/R(HBsAg-) living KTx recipients, and 83 hepatitis B core antibody (HBcAb) + living donors to HBcAb- recipients [D(HBcAb+)/R(HBcAb-)] were used as control group by reviewing medical archives and propensity score matching. Treatment failure (defined as any HBV serology conversion, liver injury, graft loss, or recipient death) is the primary endpoint.

**Results:**

Twenty-four donors (28.9%) were HBV DNA+, and 20 recipients had no HBV immunity in the D(HBsAg+)/R(HBsAg-) group pre-transplantation. HBV prophylaxis was applied in all D(HBsAg+)/R(HBsAg-) recipients, while none was applied in the D(HBcAb+)/R(HBcAb-) group. We observed a significant higher treatment failure in D(HBsAg+)/R(HBsAg-) than D(HBcAb+)/R(HBcAb-) group (21.7% vs. 10.8%, *P* < 0.001). Interestingly, no significant difference was found between groups on HBV seroconversion, liver and graft function, rejection, infection, graft loss, or death. However, 2/20 recipients without HBV immunity in the D(HBsAg+)/R(HBsAg-) group developed HBV DNA+ or HBsAg+, while none observed in the D(HBcAb+)/R(HBcAb-) group. HBV DNA+ donor and male recipient were significant risk factors for treatment failure.

**Conclusion:**

D(HBsAg+)/R(HBsAg-) should be considered for living kidney transplantation, but with extra caution on donors with HBV DNA+ and male candidates.

## Background

As the demand of organ transplantation continues to increase in the past decades, more and more medical facilities are facing serious issues including organ shortage [1–3]. This is always of great concern among transplant clinicians over the years, and it pushed the transplant centers to expand the criteria of accepting donors, including older age of 70 to 80, with significant medical history, with abnormal social behavior, or a concurrent history of hepatitis B or C virus exposure [4–8]. As reported by the World Health Organization in 2019, 257 million people were living with chronic hepatitis B virus (HBV) infection, defined by hepatitis B surface antigen positive (HBsAg+). Meanwhile, the prevalence of HBV infection rate varies among the world, with the lowest 0.7% in Americas, and the highest 6.2% in the western pacific [9]. Moreover, with the 6.2% potential donors in China were HBsAg+, proper utilization of these organs may provide undeniable benefit [10]. But, the concern of transmitting HBV infection to the recipients has never been relieved. Thus, the previous clinical practices on HBsAg+ donors were limited to HBsAg+ recipients which restricted their great use [11].

With the rapid development on HBV vaccinations, hepatitis B immunoglobulin (HBIG), and anti-viral prophylaxis treatment, i.e. nucleotide analogs, lamivudine, adefovir, entecavir et al., it may provide an effective, as well as a safe option for HBsAg+ donor organs transplanted into HBsAg- recipients [D(HBsAg+)/R(HBsAg-)] [6]. A few studies reported the efficacy and safety outcomes of D(HBsAg+)/R(HBsAg-) previously. Yilmaz et al. compared the long-term outcomes in HBsAg- kidney transplant (KTx) recipients receiving a kidney from HBsAg+ or HBsAg- donors. They found that the rate of acute hepatitis was significantly higher in recipients of HBsAg+ donors (11.5% vs. 0%). Interestingly, all patients developed acute hepatitis had acquired immunity after HBV vaccination, while patients who had natural immunity against HBsAg did not develop any acute hepatitis [4]. A recent study compared the outcomes of KTx between HBsAg- recipients with anti-HBs titer above 100 mIU/mL receiving HBsAg+ donors without HBV viremia and HBsAg- donors. With a mean follow-up of 58.2 months, researchers found no significant differences in graft and recipients’ survivals, nor HBV-infective markers (including HBsAg, HBcAb, HBeAg, HBV DNA et al.). Surprisingly, recipients of HBsAg+ donors with no prophylaxis had similar outcomes with those treated with lamivudine alone or lamivudine and HBIG. This study, therefore, suggested that KTx from HbsAg+ donors to HbsAg- recipients with protective anti-HBs titer may provide comparable graft and patient survival without HBV transmission [12].

In these respects, the 2017 *KDIGO Clinical Practice Guideline on the Evaluation and Care of Living Kidney Donors* recommended that HBsAg+ donors may be considered for HBsAg- recipients with HBV protective immunity with informed consent of the recipient, and possible antiviral HBV treatment of the recipient and post-transplant monitoring may also needed [13]. However, according to *U.S. Organ Procurement Transplant Network (OPTN) 2017 Annual Data Report: Kidney*, no D(HBsAg+)/R(HBsAg-) KTx was performed [14]. Moreover, under the circumstance that the donor is HBV DNA+, most transplant centers declined the organ due to scarce evidences on this specific topic [10]. We reported our data regarding KTx from HBsAg+ donors to HBsAg- recipients with/without HBV immunity [15]. We found that the liver and graft function, rejection rate, infection, and graft loss were comparable between D(HBsAg+)/R(HBsAg-) and D(HBcAb+)/R(HBcAb-) groups, except recipient deaths were more frequent in the D(HBsAg+)/R(HBsAg-) group. However, due to the limited positive cases, defined as HBsAg+ and HBV DNA+, it is hard to identify the associated risk factors which guide clinical decisions and evaluate the prognosis outcomes using a single event as the endpoint. Under this circumstance, a composite endpoint (multiple events all treated as one endpoint) may provide additional information on risk factors while less participants and events are required [16, 17]. Therefore, we aimed to explore the related risk factors by retrospectively analyzing the data of our single-center living donor D(HBsAg+)/R(HBsAg-) KTx using a composite endpoint named treatment failure.

## Methods

### Data collection

Every pair of living kidney donation and transplantation was approved by the Institutional Review Board of West China Hospital, Sichuan University and the Health Commission of Sichuan Province, China. And, this study protocol was reviewed and approved by the Biomedical Ethics Committee of West China Hospital (No. 2019SHEN1179).

Recipients of D(HBsAg+)/R(HBsAg-) were informed about the potential risks of HBV transmission and benefits of KTx, and written informed consents were obtained pre-surgically. Living D(HBsAg+)/R(HBsAg-) KTx performed in the West China Hospital, from January 1, 2009 to June 30, 2017 were identified retrospectively using electronic medical archives. We excluded D(HBsAg+)/R(HBsAg-) KTx when i) pre-transplant hepatitis C virus infection existed (donors and/or recipients); ii) ABO incompatible KTx; or iii) deceased donor transplantation.

It is known that D(HBsAg+)/R(HBsAg-) and HBsAg−/HBcAb+ donors to HBsAg−/HBcAb- recipients [D(HBcAb+)/R(HBcAb-)] are the most two possible sources of donor-derived HBV infection in the organ transplantation setting, and those two settings are also being discussed in the current guideliones [10, 13]. Recently, researchers found that D(HBcAb+)/R(HBcAb-) KTx, especially when the recipients are hepatitis B surface antibody positive (HBsAb)+, is at little risk of transmitting HBV infection, and the risk of graft failure or morbidity is similar to those who received non-HBV kidneys [13]. In the current study, we set the control group as D(HBcAb+)/R(HBcAb-), in order to make it closer to the real clinical setting. Propensity score matching analysis was used to match a set of measured covariates between groups, including donor/recipient sex and age, and the pre-transplant recipients’ hepatitis B surface antibody titers (HBsAb) (< 10 IU/L, 10–100 IU/L, > 100 IU/L). The 1:1 nearest neighbor matching algorithm was used on experimental and control groups.

We reviewed donors and recipients’ electronic medical records and extracted the donor/recipient demographics, end stage renal failure causes, prior transplant history, immunological features, induction, and immunosuppression regimens et al. HBV-associated parameters such as pre- and post-transplant status of HBsAg, HBsAb, hepatitis B e antigen (HBeAg), hepatitis B e antibody (HBeAb), HBcAb, HBV DNA, peri-surgical treatment including application of HBIG and antiviral prophylaxis, and liver function were also recorded upon every visit. Rejection, graft function, graft loss, and recipient death were also analyzed. Clinical assessment, HBV serology and DNA were examined when the recipient did not show any post-transplant HBV seroconversion at the last follow-up visit (December, 2017).

### Statistical analysis

The primary endpoint was the treatment failure rate. We defined the primary endpoint as a combination of treatment failure: post-transplant HBV DNA- → +, HBsAg- → +, HBeAg- → +, HBeAb- → +, HBcAb- → +, clinical liver injury, graft loss, or recipient death (whichever was reached first), followed by the rules published by Dr. McCoy [18]. HBV DNA was measured by using fluorescent real-time polymerase chain reaction (RT-PCR), Roche COBAS® TaqMan® HBV Test. HBsAb titers were graded as following criteria: negative (< 10 IU/L), positive (10–100, 100–1000, > 1000 IU/L). Any rises to the titer grade, i.e. < 10 → 10–100 IU/L, 100–1000→ > 1000 IU/L, were considered an upgrade, while any decreases to the titer grade, i.e. > 1000 → 100-1000 IU/L, 10–100 → < 10 IU/L, were considered a downgrade. Liver function of the recipients was examined using biochemical test of the serum, and normal liver function was defined as serum alanine aminotransferase (ALT) below 40/50 IU/L (female/male), or total bilirubin < 28 μmol/L. Active liver injury was defined as ALT > 80/100 IU/L (female/male), or total bilirubin > 34 μmol/L.

Baseline characteristics of the experimental and control groups were compared by using Student’s t, Chi-square or Wilcoxon rank sum tests when appropriate. Chi-square test was used to investigate the differences of post-transplant clinical complications between the two groups, and non-parametric test was applied in laboratory parameter comparison. Graft and patient survival rates were estimated using the Kaplan-Meier method, and the differences in survival rates were compared using the log-rank test univariately. To explore the risk factors significantly associated with treatment failure, experimental group recipients not reaching treatment failure were used as reference, and univariate and multivariate logistic regression was utilized to screen potential risk factors related to treatment failure. Variables in the regression models with the lowest Akaike information criterion (AIC) and lowest Bayesian information criterion (BIC) were selected as the significant factors, and odds ratio (OR) and 95% confidence interval (CI) were calculated by logistic regression. All statistical analyses were conducted by R version 3.6.1, with *p*<0.05 considered as statistically significant.

## Results

### Baseline characteristics

From January 1st, 2009 to June 30th, 2017, 83 D(HBsAg+)/R(HBsAg-) and 384 D(HBcAb+)/R(HBcAb-) KTx patients were identified from 2071 living donor KTx in our transplant center, with a percentage of 4.0 and 18.5%, respectively. After propensity score matching 83 D(HBcAb+)/R (HBcAb-) were included in the control group. The baseline demographic, clinical, and immunological data of both groups, including donor and recipient, are summarized in Table 1. All baseline characters were comparable in both groups, including age, gender, cause of end stage renal failure, preemptive transplant rate, duration of dialysis, panel reactive antibody, induction therapy and initial immunosuppressants, except for a higher HLA mismatch was found in the D(HBsAg+)/R (HBsAg-) group (*p* = 0.004). No evidences of abnormal liver enzymes, total bilirubin, coagulation dysfunction, or liver cirrhosis were noticed before surgery.

**Table 1:** Baseline demographic, clinical and immunological characteristics in the two groups

	D(HBsAg+)/R(HBsAg-) group (*n* = 83)	D(HBcAb+)/R(HBcAb-) group (*n* = 83)	*P* value
Donor			
Median age, year (range)	50 (31–66)	48 (28–66)	0.133
Male (%)	39 (47.0)	40 (48.2)	1.000
Living related (%)	83 (100)	83 (100)	/
Recipient			
Median age, year (range)	32 (9–51)	29 (15–51)	0.093
Male (%)	64 (77.1)	64 (77.1)	1.000
Cause of end stage renal failure			
Glomerulonephritis (%)	40 (48.2)	26 (31.3)	0.084
Non-glomerulonephritis (%)	8 (9.6)	10 (12.0)	
Unknown (%)	35 (42.2)	47 (56.6)	
Preemptive transplant (%)	6 (7.2)	9 (10.8)	0.588
Median duration on dialysis, months (range)	9 (0–120)	8 (0–84)	0.497
Mean HLA mismatch (A, B, DR, DQ)	4.04 ± 1.47	3.46 ± 1.07	**0.004**
PRA > 0(%)	27 (32.5)	22 (26.5)	0.496
Second transplant	0	0	/
Induction therapy			
IL-2 receptor antagonist (%)	49 (59.0)	55 (66.3)	0.553
Antithymocyte globulin (%)	18 (21.7)	13 (15.7)	
No induction (%)	16 (19.3)	15 (18.1)	
Initial immunosuppression			
Tac + MPA + Pred (%)	78 (94.0)	80 (96.4)	0.469
CsA + MPA + Pred (%)	5 (6.0)	3 (3.6)	

*CsA* cyclosporin A, *HLA* human leukocyte antigen*, MPA* mycophenolic acid, *PRA* panel reactive antibody*, Pred* prednisone, *Tac* tacrolimus

### Pre-transplant and post-transplant HBV status

HBV serology parameters of the experimental and control groups pre- and post-transplantation were summarized in Table 2. We identified 24 pre-transplant HBV DNA+ donors in the experimental group, and their pre-transplant median HBV DNA level was 1.20 × 10^3^ IU/ml (range 5.86 × 10–4.04 × 10^6^). In the control group, we did not use any prophylaxis treatment of HBV. Meanwhile, in the experimental group, all recipients received prophylaxis treatment as following: HBIG alone (*n* = 18, 21.7%), antiviral alone (*n* = 41, 49.4%), and combination of HBIG and antiviral (*n* = 24, 28.9%). HBIG was infused as a single dose of 2000 IU pre-transplantation, and antiviral treatment started on the first day post-transplantation. Among the 65 recipients (78.3%) who received antiviral prophylaxis, 49 received lamivudine, whereas the other 16 were on entecavir. Antiviral treatment duration was 1–3 months (due to the nature of retrospective study, the exact duration cannot be provided).

**Table 2:** Pre/post-transplant HBV serology and post-transplant complications in the two groups

	D(HBsAg+)/R(HBsAg-) group (*n* = 83)	D(HBcAb+)/R(HBcAb-) group (*n* = 83)	*P* value
Donors’ pre-transplant HBV serology			
HBsAg+	83 (100%)	0	**< 0.001**
HBsAb+	3 (3.6%)	58 (69.9%)	**< 0.001**
HBeAg+	0	0	/
HBeAb+	78 (94.0%)	35 (42.2%)	**< 0.001**
HBcAb+	82 (98.8%)	83 (100%)	1.000
HBV DNA+	24 (28.9%)	Unknown^a^	/
Recipients’ pre-transplant HBV serology			
HBsAg+	0	0	/
HBsAb+	63 (75.9%)	63 (75.9%)	1.000
HBsAb titer > 100	32 (38.6%)	32 (38.6%)	1.000
HBeAg+	0	0	/
HBeAb+	34 (41.0%)	0	**< 0.001**
HBcAb+	58 (69.9%)	0	**< 0.001**
HBV DNA+	0	Unknown^a^	/
Recipients’ most recent HBV serology			
HBV DNA - → +	2^b^ (2.4%)	0	0.477
HBsAg - → +	2^b^ (2.4%)	0	0.477
HBeAg - → +	1 (1.2%)	0	1.000
HBeAb - → +	4 (4.8%)	0	0.129
HBeAb + → -	4 (4.8%)	0	0.129
HBcAb - → +	7 (8.4%)	2 (2.4%)	0.170
HBsAb titer downgrade	1 (1.2%)	11 (13.3%)	**0.012**
HBsAb titer upgrade	13 (15.7%)	2 (2.4%)	**0.013**
Recipients’ post-transplant complications			
Treatment failure	18 (21.7%)	9 (10.8%)	**< 0.001**
Delayed graft function	2 (2.4%)	0	0.477
Rejection	12 (14.5%)	12 (14.5%)	1.000
Infection	34 (41.0%)	22 (26.5%)	0.071
Graft loss	4 (4.8%)	4 (4.8%)	1.000
Recipient death	5 (6.0%)	1 (1.2%)	0.216
Abnormal liver function	29 (34.9%)	32 (38.6%)	0.650
Active liver injury	8 (9.6%)	2 (2.4%)	**0.048**
Malignancy	0	0	/

^a^We did not test the pre-transplant HBV DNA levels of the donors or recipients in the D(HBcAb+)/R(HBcAb-) group

^b^ Two recipients in the D(HBsAg+)/R(HBsAg-) group developed post-transplant HBV DNA+ accompanied with HBsAg+. The first recipient: 32-year-old male, pre-transplant donor/recipient HBV serology was HBV DNA −/−, HBsAg +/−, HBsAb −/−, HBeAg −/−, HBeAb +/−, HBcAb +/−. His prophylaxis was lamivudine alone for 1.5 months. The recipient experienced a temporary rise of ALT, up to 163 IU/L 1 year after transplantation. Then he was lost to follow-up during post-transplant years 1.5–5.5. He was found to have HBV DNA+ (5.02 × 10^4^ IU/ml), HBsAg+, HBeAb+, and HBcAb+, ALT 55 IU/L when admitted due to pulmonary infection 5.5 years after transplantation. He received long-term entecavir monotherapy until he died of pulmonary infection 7 years after transplantation. The second recipient: 24-year-old male, pre-transplant donor/recipient HBV serology was HBV DNA + (1.14 × 10^3^ IU/mL)/−, HBsAg +/−, HBsAb −/−, HBeAg −/−, HBeAb +/−, HBcAb +/−. His prophylaxis was HBIG 2000 IU and lamivudine for 2 months. He received a total of 1500 mg Intravenous methylprednisolone to treat acute rejection 5 months after transplantation. He became HBV DNA+ (> 5.00 × 10^7^ IU/mL), HBsAg+, and HBcAb+ 6 months after transplantation. His total bilirubin level rapidly increased from 26 μmol/L at month 6 to 489 μmol/L at month 8 when he died of liver failure and pulmonary infection

Two recipients in the experimental group showed seroconversion evidences: HBV DNA- to +, and HBsAg- to +, but none in control group, with a median follow-up of 36 months (range, 6–106 months) for the experimental group, and 36 months (range, 4–107 months) for the control group (Table 2).

### Post-transplant clinical outcomes and laboratory parameters

Post-transplant clinical complications indicated that the experimental group had a higher incidence of treatment failure and active liver injury rate than control group (Table 2). Most of the post-transplant laboratory parameters were comparable, except that the experimental group had lower total bilirubin level at 24 months post-transplant (*p* = 0.021) (Table 3). Both groups had no significant differences on graft survival rate at 1 (98.8% vs. 100%, *p* = 0.17), 3 (97.6% vs. 96.4%, *p* = 0.84), and 5 years (97.6% vs. 95.2%, *p* = 0.62), and no significant differences in patient survival rate at 1 (97.6% vs. 98.8%, *p* = 0.15), 3 (97.6% vs. 98.8%, *p* = 0.79), and 5 years (95.2% vs. 98.8%, *p* = 0.68) was noticed.

**Table 3:** Laboratory parameters of two groups at different timepoints after kidney transplantation

	D(HBsAg+)/R(HBsAg-) group (*n* = 83)	D(HBcAb+)/R(HBcAb-) group (*n* = 83)	*P* value
**1 month**			
Alanine aminotransferase (IU/L)	25.69 ± 20.93	26.76 ± 17.22	0.377
Total bilirubin (μmol/L)	7.86 ± 3.04	8.44 ± 3.24	0.152
Serum creatinine (μmol/L)	110.18 ± 35.76	113.15 ± 36.44	0.490
eGFR (ml/min/1.73m^2^)	70.75 ± 28.73	68.46 ± 20.95	0.805
**3 months**			
Alanine aminotransferase (IU/L)	23.49 ± 17.99	23.72 ± 18.49	0.695
Total bilirubin (μmol/L)	9.35 ± 3.90	9.63 ± 3.50	0.342
Serum creatinine (μmol/L)	112.28 ± 31.89	111.61 ± 29.62	0.718
eGFR (ml/min/1.73m^2^)	67.17 ± 20.90	68.21 ± 20.65	0.968
**6 months**			
Alanine aminotransferase (IU/L)	28.33 ± 29.77	22.54 ± 16.98	0.261
Total bilirubin (μmol/L)	11.74 ± 4.97	12.19 ± 4.97	0.577
Serum creatinine (μmol/L)	109.37 ± 30.24	108.89 ± 32.48	0.966
eGFR (ml/min/1.73m^2^)	69.15 ± 21.32	71.30 ± 22.68	0.527
**12 months**			
Alanine aminotransferase (IU/L)	25.93 ± 25.88	18.60 ± 11.93	0.136
Total bilirubin (μmol/L)	18.52 ± 53.65	13.21 ± 5.60	0.327
Serum creatinine (μmol/L)	123.33 ± 126.84	108.65 ± 39.54	0.461
eGFR (ml/min/1.73m^2^)	68.71 ± 25.50	71.50 ± 21.48	0.213
**18 months**			
Alanine aminotransferase (IU/L)	23.27 ± 22.19	22.62 ± 19.14	0.566
Total bilirubin (μmol/L)	12.54 ± 5.84	13.60 ± 6.88	0.220
Serum creatinine (μmol/L)	120.06 ± 114.72	106.99 ± 33.75	0.902
eGFR (ml/min/1.73m^2^)	69.66 ± 21.99	71.91 ± 21.68	0.699
**24 months**			
Alanine aminotransferase (IU/L)	21.21 ± 15.74	20.11 ± 16.06	0.310
Total bilirubin (μmol/L)	11.35 ± 4.83	13.70 ± 6.35	**0.021**
Serum creatinine (μmol/L)	110.48 ± 35.65	118.67 ± 70.09	0.981
eGFR (ml/min/1.73m^2^)	68.11 ± 21.36	69.08 ± 22.93	0.617
**30 months**			
Alanine aminotransferase (IU/L)	20.43 ± 14.91	22.04 ± 14.93	0.573
Total bilirubin (μmol/L)	12.13 ± 5.01	13.65 ± 7.95	0.605
Serum creatinine (μmol/L)	106.22 ± 35.18	110.57 ± 50.90	0.939
eGFR (ml/min/1.73m^2^)	70.83 ± 23.80	70.80 ± 21.65	0.696
**36 months**			
Alanine aminotransferase (IU/L)	19.00 ± 15.36	23.63 ± 17.09	0.087
Total bilirubin (μmol/L)	12.37 ± 4.73	12.72 ± 5.74	0.936
Serum creatinine (μmol/L)	102.63 ± 37.91	119.54 ± 97.28	0.291
eGFR (ml/min/1.73m^2^)	72.57 ± 22.90	70.39 ± 21.79	0.874

*eGFR* estimated glomerular filtration rate

### Risk factors of treatment failure in the D(HBsAg+)/R(HBsAg-) group

To address the risk factors of treatment failure of D(HBsAg+)/R(HBsAg-) KTx on HBV infection, the living donors’ and corresponding recipients’ pre-transplant HBV status and post-transplant treatment failure in the D(HBsAg+)/R(HBsAg-) group were therefore analyzed. From a clinical prospective, the definition of treatment failure would most likely to be HBV transmission (infection evidences), graft loss, severe complications et al. After reviewing recipients’ data, we observed a low rate of HBV DNA/HBsAg/HBeAg – to +, graft loss, clinical liver injury and death of the recipient, which make it inaccurate to elucidate any risk factors. Thus, we expanded our criteria to any serology evidence related to HBV, including HBV related antibody change. Potential factors were analyzed for the prognostic value of treatment failure including pre-transplant donor HBV DNA- vs +, pre-transplant recipient factors (including age, sex, HBsAb, HBeAb, and HBcAb status), HBV prophylactic regimens (including HBIG, antiviral treatment etc.). Logistic regression models were generated, and each model was evaluated by AIC and BIC. The logistic regression results demonstrated that pre-transplant HBV DNA+ donor and male recipient were the only two significant risk factors for treatment failure of recipients, on the contrary, pre-transplant HBcAb+ of the recipients was the only significant protective factor (Tables 4 and 5).

**Table 4:** The pre-transplant HBV status of the living donors and corresponding recipients and post-transplant treatment failure in the D(HBsAg+)/R(HBsAg-) group

HBsAg+ donor^a^					HBsAg- recipients^b^				Treatment failure								
HBsAb	HBeAb	HBcAb	HBV DNA	No	HBsAb	HBeAb	HBcAb	No	HBV DNA - → +	HBsAg - → +	HBeAg - → +	HBeAb - → +	HBcAb - → +	Clinical liver injury	Graft loss	Recipient death	Total
-	+	+	-	55	+	+	+	20						1	1		1
					+	-	+	15						1		1	1
					+	+	-	1					1				1
					+	-	-	8				2	2	1			3
					-	+	+	2									0
					-	-	+	3									0
					-	-	-	6	1	1		1	1	1	1	2	3
-	+	+	+	22	+	+	+	7						1	1	1	2
					+	-	+	5				1					1
					+	-	-	4					2				2
					-	+	+	3						1	1		2
					-	-	-	3	1	1	1		1	2		1	2
-	-	+	-	3	+	-	-	1									0
					-	-	-	2									0
+	+	+	+	1	+	-	+	1									0
+	-	+	-	1	-	+	+	1									0
+	-	-	+	1	+	-	+	1									0

^a^ All HBsAg+ living donors were HBeAg- before donation

^b^ All HBsAg- recipients were HBeAg- and HBV DNA- before transplantation

**Table 5:** Factors for post-transplant treatment failure in the D(HBsAg+)/R(HBsAg-) group

Logistic regression models	Factors for treatment failure	Status	No. recipients with treatment failure/ total recipients	OR(95% CI)
Model A^a^	Pre-transplant donor HBV DNA	Negative	9/59 (15.3%)	1
		Positive	9/24 (37.5%)	6.73 (1.62,36.47)
	Recipient sex	Female	1/19 (5.3%)	1
		Male	17/64 (26.6%)	16.65 (2.07,397.13)
	Pre-transplant recipient HBcAb	Negative	11/25 (44.0%)	1
		Positive	7/58 (12.1%)	0.08 (0.02,0.31)
	Anti-viral prophylaxis	No	4/18 (22.2%)	1
		Yes	14/65 (21.5%)	0.66 (0.13,3.43)
Model B^b^	Pre-transplant donor HBV DNA	Negative	9/59 (15.3%)	1
		Positive	9/24 (37.5%)	5.95 (1.55,29.39)
	Recipient sex	Female	1/19 (5.3%)	1
		Male	17/64 (26.6%)	17.10 (2.14,407.15)
	Pre-transplant recipient HBcAb	Negative	11/25 (44.0%)	1
		Positive	7/58 (12.1%)	0.08 (0.02,0.31)

*OR* odds ration*, CI* confidence interval

^a^ Lowest Akaike information criterion (71.11)

^b^ Lowest Bayesian information criterion (80.94)

## Discussion

In order to expand the donor pool, numerous efforts have been made by researchers and clinicians. Testing the feasibility of potential infected donors has always been a hot area. The 2018 British guidelines for living donor KTx recommended that active HBV infection of the donor is considered as a contraindication for living kidney donation [19]. However, a few attempts have been made on D(HBsAg+)/R(HBsAg-) KTx [12, 20]. Jiang et al.’s study recruited 65 HBsAb+ recipients, and found only 2 recipients developed de novo HBsAg+, while no patient developed severe liver dysfunction nor died [20]. Dr. Chancharoenthana et al. included 43 HBsAb titer > 100 IU/L recipients, and no evidences was found on donor derived HBV transmission [12]. While these encouraging clinical outcomes help clinicians push the limit, the practice is still not generally accepted. A recent survey showed that only 35% transplant clinicians suggested donor with HBV was acceptable with proper prophylaxis, while the other 44% declined [19]. Our results confirmed the safety of D(HBsAg+)/R(HBsAg-) KTx in immunized recipients as there was no HBsAg+ nor HBV DNA+ found in HBSAb+ recipients. Interestingly, 4 recipients developed de novo HBeAb and 7 developed HBcAb in D(HBsAg+)/R(HBsAg-) recipients after KTx. The etiology of the detection of HBeAb and HBcAb in previously HBV unexposed recipients remains unclear [21]. To some extent, the HBV seroconversion indicated HBV transmission occurred, but it seems the infection did not caused any serious consequences [22].

Only a few cases of donor-derived HBV transmission have been previously reported in HBsAb+ recipients [12]. In our study, 2 cases of de novo HBsAg+ or HBV DNA+ were observed in HBsAg- recipients, while none in the HBsAb+ recipients. Therefore, HBV vaccination should be highly recommended pre-surgically for KTx candidates. However, the HBV vaccination in dialysis patients is not as effective as healthy people, with approximately 48.6% non-responders [23]. Moal et al. analyzed HBV serology change of KTx recipients. They found that nearly 25% of the general KTx population would lose protective HBsAb titers after 12 months [24]. Contrarily, we observed downgrades and upgrades of post-transplant HBsAb titer in 1/83 (1.2%) and 13/83 (15.7%) of D(HBsAg+)/R(HBsAg-) recipients, compared to 11/83 (13.3%) and 2/83 (2.4%) of D(HBcAb+)/R(HBcAb-) recipients. This indicated that D(HBsAg+)/R(HBsAg-) KTx might act like an HBV “vaccination”. Moreover, we reported a higher incidence of treatment failure and active liver injury in D(HBcAb+)/R(HBcAb-) group, along with higher HLA mismatches. As known, HLA mismatch increases graft dysfunction and shorten graft survival [25–27]. To further address this issue, we also performed Cochran-Mantel-Haenszel test stratified by HLA mismatch. There was still a higher prevalence of active liver injury in the experimental group than in the control group. Therefore, D(HBsAg+)/R (HBsAg-) is still considered to be the primary cause for the higher incidence of active liver injury.

To further reduce the transmission risk of HBV in KTx recipients, a proper prophylaxis is essential [10]. With the various regimens available on the market right now, ie. vaccine, HBIG and several antiviral drugs, no consensus on the optimal prophylaxis have been made [10]. Berber et al. reported no HBV transmission occurred using 1–3 year lamivudine in HBsAb+ recipients from HBsAg−/ unknown HBeAg and HBV DNA deceased donors [28]. Jiang et al.’s used a grading prophylaxis: All recipients receive HBIG 400 IU on transplant day and 1 month after. If the donor was HBV DNA+, the recipient was given HBIG 400 IU weekly for 3 months, and lamivudine 100 mg per day for 6 consecutive months [20]. Tuncer et al.’s protocol used no prophylaxis when recipient has HBsAb. And HBV vaccinations were used to increase titers when HBsAB less than 10 IU/L. They declared no de novo HBV infection in 2 years post-transplantation [29]. Chancharoenthana et al.’s study found no difference of D(HBsAg+/no HBV viremia)/R(HBsAg−/HBsAb> 100 IU/L) KTx whether prophylaxis (lamivudine, HBIG, or combination) used or not. Magiorkinis et al. reported a case of HBsAb titer was 11.6 IU/L, and received HBIG and HBV vaccine, but no antiviral prophylaxis died after KTx [30]. Based on these heterogeneous evidences, it is now recommended that non-liver recipients who are HBsAb- and HBcAb- to take antiviral prophylaxis for up to 1 year [31]. However, further studies are still required to develop the optimal prophylaxis protocol.

HBV is reported have huge negative impact on recipient survival. Chen et al. reported that the 1, 3, 5, and 10 years patients survival were lower for patient with HBV activation compared to those without [32]. Positive on serology HBsAb with HBcAb cannot assure fully protection on HBV transmission. Chen et al. also reported 13.3% in HBsAb+/HBcAb+ recipients experienced HBV activation compared to 42% in the HBsAb−/HBcAb+ group [32]. Thus, it is important to address the relative risk factors for HBV activation or transmission. However, few previous studies explored risk factors of donor-derived HBV transmission. Chen et al’s logistic regression demonstrated HBsAb and prophylaxis (lamivudine) were independent protective factors, while older age (> 60 years old) and anti-T cell immunosuppressants were risk factors of HBV activation [32]. To identify all potential related risk factors related to HBV transmission and survival with limited clinical data, a composite endpoint were used in the present study. The purported benefits of composite endpoint including increased statistical efficiency, decrease in sample-size requirements, and shorter trial duration [18]. The logistic regression models showed both pre-transplant HBV DNA+ donor and male recipients were independent risk factors of treatment failure, and pre-transplant HBcAb+ of the recipient was a protective factor. These results indicated that donor and recipient factors are more important than the application of HBV prophylaxis for treatment failure. Recipients carrying one or more risk factors of treatment failure should be closely monitored for possible risk of HBV transmission and should receive more intensive HBV prophylaxis. Out of our expectation, it is not the pre-transplant recipient HBsAb+ but HBcAb+ a protective factor of treatment failure in our study. In 58 HBcAb+ recipients, 49 recipients (84.5%) were also HBsAb+. Among all 83 recipients, 5 (10.2%) in 49 HBsAb+/HBcAb+ recipients, and 6 (42.9%) in 14 HBsAb+/HBcAb- showed treatment failure, which might indicate that natural immunity (HBsAb+/HBcAb+) is more protective than vaccine immunity (HBsAb+/HBcAb-). Dr. Baig reported a male dominance in all categories of HBV infected patients [33]. As a recent survey also conclude that male HBsAg+ more dominant than female, and, an HBsAg+ of roughly 14% was found in middle-aged males, while 6.2% in females [34]. Furthermore, several studies looked in to the mechanisms of sexual disparities. Yang et al. identified a unique protein named apolipoprotein A-I in male, which sought to be the reason of HBV infection and related complications [35]. Some researchers also shown that sex hormones are playing a crucial role in the progression of HBV infection and the development of HBV related hepatocellular carcinoma [36, 37]. These evidences might explains why male recipients are at a higher risk of HBV transmission. As well known, the presence of HBV DNA in serum of donors is a reliable marker of active HBV replication. Though various prophylaxis options are available now, it seemed still not enough in protecting the recipient without HBsAb. Hence, the risk of transmitted HBV infection from HBV active infected donors should be informed to the candidates prior to surgery.

Our study is the largest cohort of D(HBsAg+)/R(HBsAg-) KTx to date, including 83 living D(HBsAg+)/R(HBsAg-) KTx cases. And, the present study also represents the largest cohort of D(HBsAg+)/R(HBsAg-) KTx till now, with a 2.4% rate of treatment failure using HBV prophylaxis. Our data should also be carefully interpreted. Instead of using D(HBcAb-)/R(HBcAb-), we choose to use D(HBcAb+)/R(HBcAb-) as control group. Though there are studies and guidelines saying D(HBcAb+)/R(HBcAb-) has similar clinical outcomes, it is still thought to be riskier than D(HBcAb-)/R(HBcAb-). Therefore, some positive outcomes may be undiscovered by the current setting. In addition, we also used a composite endpoint in the current study. Though there are advantages, the other effects of using it should also be noticed. When some of the endpoints have opposite effect on the composite endpoint, misleading the result may occur. And, the weight of each endpoint should also be carefully calculated. Moreover, due to the nature of retrospective study, and lacking the routine monitoring of post-transplant HBV clinical parameters, the natural history of donor-derived HBV transmission cannot be fully explored. The donors’ pre-donation antiviral treatment and recipients’ pre-transplant HBV vaccinations information was unable to retrieve, and the transmission rate may be not accurate as some could have been transiently HBsAg+ and then subsequently cleared by the recipient’s immunity or prophylaxis. Moreover, all our donors recruited in our study were HBeAg- pre-transplant, thus our findings may not be applied to HBeAg+ donors. Though risky, we transplant HBsAg+ living donor kidneys to HBsAg- or HBsAb- recipient is mainly because of the highly heterogenous immune response of vaccination among dialysis patient, and the balance between uncertain waiting time and worsen body condition [23]. Our study indicated that HBsAb- recipients were at a higher but still acceptable risk of HBV transmission. Especially when considering the survival situation in transplant recipient and those on waiting list, the decision becomes easier [38]. The worst case is HBV transmission to the recipient, and clinicians can still achieve long term HBV suppression via antiviral nucleoside therapy [39]. Therefore, informed consents, more intensive HBV prophylaxis, and post-transplant monitoring were still in great need for those willing to accept HBsAg+ kidneys [40].

## Conclusions

Compared to D(HBcAb+)/R(HBcAb-), D(HBsAg+)/R(HBsAg-) KTx had more treatment failures and active liver injuries. However, HBV seroconversion, rejection, infection, graft loss, and recipient death were not significantly different between the two groups. Owing to the organ shortage, D(HBsAg+)/R(HBsAg-) should not be a contraindication to living kidney donation. HBV DNA+ donor and male recipient were significant risk factors for treatment failure and should be treated with extra caution. Although our study provides initial evidence of the safety of transplanting HBsAg+ kidneys into HBsAg−/HBsAb- recipients, the optimal choice and duration of prevention strategies for non-immune recipients merit further study.

## Data Availability

The datasets used and/or analysed during the current study are available from the corresponding author on reasonable request.

## Abbreviations

AIC: Akaike information criterion
ALT: Alanine aminotransferase
BIC: Bayesian information criterion
CI: Confidence interval
D(HBsAg+)/R(HBsAg-): Transplantation from HBsAg+ donor to HBsAg- recipient
D(HBcAb+)/R(HBcAb-): Transplantation from HBcAb+ donor to HBcAb- recipient
HBcAb: Hepatitis B core antibody
HBeAb: Hepatitis B e antibody
HBeAg: Hepatitis B e antigen
HBIG: Hepatitis B immunoglobulin
HBsAb: Hepatitis B surface antibody
HBsAg: Hepatitis B surface antigen
HBV: Hepatitis B virus
KTx: Kidney transplantation
OPTN: Organ Procurement Transplant Network
OR: Odds ratio
